# True intratracheal oxygen concentration delivered by SentriO Oxy™ masks under various respiratory conditions: a bench study

**DOI:** 10.1007/s10877-023-01076-4

**Published:** 2023-10-13

**Authors:** Cheng Chiang, Wei-Nung Teng, Ting-Yun Chiang, Chao-Lan Huang, Shi-Pin Lin, Wen-Kuei Chang, Chien-Kun Ting

**Affiliations:** 1 Department of Anesthesiology, Taipei Veterans General Hospital and National Yang Ming Chiao Tung University, Taipei, Taiwan; 2 Institute of Emergency and intensive care medicine, National Yang Ming Chiao Tung University, Taipei, Taiwan

**Keywords:** SentriO Oxy™, Fraction of inspired oxygen, Hypoxemia, Oxygenation, Oxygen delivery device

## Abstract

SentriO Oxy™ is a newly available, Food and Drug Administration-approved oxygenation mask system that provides high oxygenation, even on low-flow (5–10 L/min) oxygen. This study aimed to accurately measure the intratracheal fraction of inspired oxygen (FiO_2_) using SentriO Oxy™ masks under relatively low oxygen flow rates. A manikin-ventilator-test lung simulation system was used. We measured FiO_2_ at the level of the carina, 5 minutes after applying 45 different respiratory parameter combinations using SentriO Oxy™ masks. Tidal volume (TV) was set to 300, 500, and 700 mL; respiratory rate (RR) was set to 8, 12, 16, 20, and 24 breaths per minute; and oxygen flow rate was set to 6, 8, and 10 L/min. Our hypothesis was that FiO_2_ would be proportional to the difference between oxygen flow rate and minute ventilation. FiO_2_ measured by smaller TV, lower RR, or higher oxygen flows revealed a significantly higher value, confirming our hypothesis. In addition, using linear regression analysis, we found that TV, RR, and oxygen flow were all significant factors influencing the measured FiO_2_. Our experiment proposed two prediction equations considering the oxygen flow rate, TV, and RR. The results of our study may provide information and prediction of FiO_2_ for clinicians to use SentriO Oxy™ masks during sedative anesthetic procedures under low oxygen flow rates.

## Introduction

Oxygen therapy is of great importance in many areas of medicine, and supplemental oxygen is indicated when hypoxemia is suspected [1, 2]. During any sedative anesthetic procedure, oxygen supplementation should be considered to decrease the incidence of hypoxemia [3]. Face masks are widely used for oxygenation. Non-rebreathing masks have a one-way valve and a reservoir bag to prevent room air entrainment and rebreathing of exhaled gases, allowing them to provide a nearly 90% fraction of inspired oxygen (FiO_2_) at a flow rate of > 30 L/min [4]. SentriO Oxy™ is a newly available, Food and Drug Administration-approved oxygenation mask system that provides high oxygenation even with low-flow (5–10 L/min) oxygen. Compared with high flow nasal cannula systems (up to 60 L/min) [5] and traditional non-rebreathing masks (10–30 L/min), it requires significantly less oxygen consumed per unit of time. According to its manufacturer, SentriO Oxy™ is superior to a traditional NRM because it has a proprietary multi-valved controller manifold which preferentially delivers all available oxygen to the alveolar regions of the lungs, and generally fills the anatomical dead space (ANS) with ambient air if and when oxygen supply is mismatched with peak inspiratory flow. However, the actual oxygen concentration that can be utilized by patients is currently unknown. Previous studies have attempted to measure the oxygen concentration in oxygenation systems other than the SentriO Oxy™ in living human participants [6, 7]; however, variations between patients and within patients are not negligible, and it is difficult to compare the results of different studies. Some researchers have measured the FiO_2_ using a catheter placed behind the uvula, in efforts to extract a gas sample [8]. However, the exact intratracheal oxygen concentration is difficult to measure non-invasively, with the exception of tracheostomized patients [9]. We conducted this bench study to investigate the true FiO_2_ using SentriO Oxy™ masks, using a manikin-test lung-ventilator system simulating the spontaneous breathing cycle. The simulation system has been introduced before [10–12], and minor modifications were made to better mimic real-life scenarios. The purpose of this study was to measure the accurate intratracheal FiO_2_ using SentriO Oxy™ masks under relatively low oxygen flow rate. Additionally, we hypothesized that FiO_2_ would be proportional to the difference between the oxygen flow rate and minute ventilation delivered by the ventilator.

## Methods

A test lung (Dual Adult TTL Lung, Michigan Instruments, 4717 Talon Court SE, Grand Rapids, MI 49,512 USA) with two independent bellows, linked with a rigid metal coupling clip, was used. The driving bellow was connected to a Dräger ventilator (Primus® Anaesthesia Workstations, Drägerwerk AG & Co. KGaA, Moislinger Allee 53–55, 23,558 Lübeck, Germany), and the other bellow was connected to the manikin’s trachea (AirSim Advance X, Product Code: AA91100X, Tru Corp, 33 Waringstown Road Lurgan, Co.Armagh, N. Ireland, BT667HH), mimicking the correct anatomy of an adult airway and face contour. An oxygen rotameter was applied to the SentriO Oxy™ mask (HealO Medical, LLC and HealOMed Scientific, Inc.) to deliver oxygen at the rate of 6 to 10 L/min as per the manufacturer’s instructions. The mask was then gently applied on the manikin’s face as seamlessly as possible. When the ventilator delivered a tidal volume (TV), the driving bellow expanded and forced the metal strap to pull the other bellow, thereby stimulating a spontaneous breath by creating negative pressure and absorbing gas through the manikin to the trachea. The compliance of the test lung was set to 50 mL/cm H_2_O, while the Inspiratory-to-expiratory time ratio was set to 1:2, to represent normal breathing physiology. A TV range of 300–700 mL and respiratory rate (RR) range of 8 to 20 breaths per minute were set to represent various breathing patterns. The gas sampled from the manikin’s trachea was analyzed by the ventilator automatically and continuously, and the sample rate was 150 ± 20 mL per minute. Oxygen concentration measurements were obtained at equilibrium by the gas analyzer integrated into the ventilator. This equilibrium was assumed when the reading remained constant for 5 min. After completing each experiment, fresh gas was delivered to wash out the excess oxygen in the model until the measured FiO_2_ reached 21%. Each parameter setting was tested five times to eliminate possible errors.

**Fig. 1:**
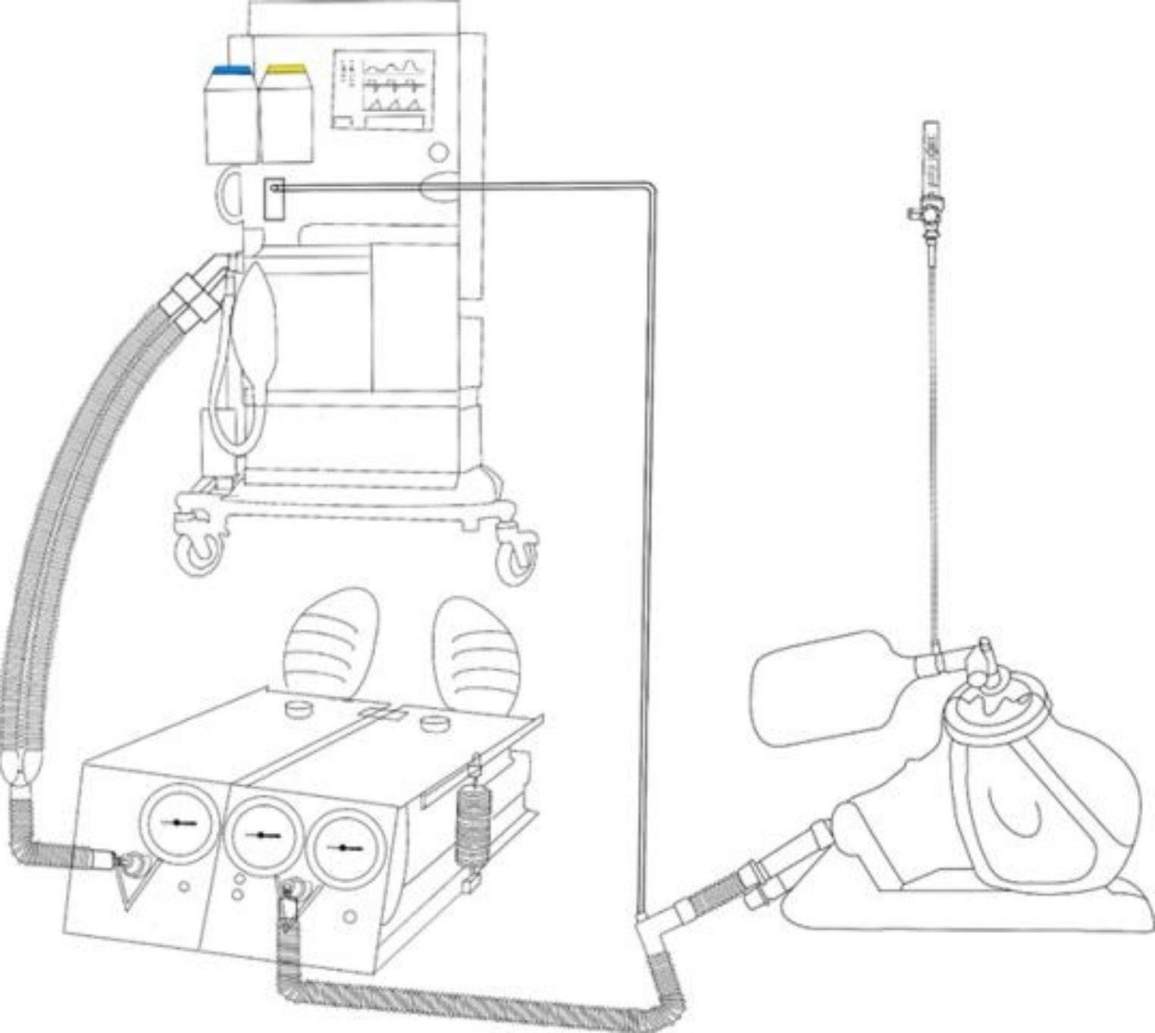
Experimental apparatus of the test lung model

The FiO_2_ was determined 5 min after the ventilator was turned on. A total of 225 samples were collected in this study. The means and standard deviations (SD) were calculated within the repeated samples under the same parameters. The whole setting of the system is shown in Fig. 1. A Kruskal–Wallis test with a post-hoc Bonferroni was performed to test between-group differences. To test the predictive factors associated with FiO_2_, multiple linear regression model analysis was performed to examine whether TV, RR, and oxygen flow rate were significant predictors. Statistical analysis was performed using R programming language, version 4.2.0. A p-value of < 0.05 was considered statistically significant.

## Results

The SentriO Oxy™ mask was tested with 15 unique combinations of TV and RR, in addition to three different oxygen flow rates. All combinations and corresponding FiO_2_ were listed in Table 1. Kruskal–Wallis test with a post-hoc Bonferroni was performed in different comparisons while TV and oxygen flow were controlled, or when TV and RR were controlled. A total of 24 comparisons were conducted, with individual p values listed, which all reached statistically significance.

**Table 1:** Measured FiO_2_ under different parameter settings

Flow^1^	RR	TV	FiO2	Flow	RR	TV	FiO2	Flow	RR	TV	FiO2	*P* ^*3*^
6	8	300	95.0 ± 0.837	6	8	500	89.4 ± 2.408	6	8	700	84.2 ± 1.924	*0.002402*
6	12	300	87.2 ± 1.643	6	12	500	80.2 ± 2.280	6	12	700	75.2 ± 1.924	*0.002111*
6	16	300	82.4 ± 2.510	6	16	500	72.6 ± 0.894	6	16	700	70.2 ± 2.280	*0.003783*
6	20	300	77.8 ± 1.789	6	20	500	68 ± 2.915	6	20	700	63 ± 1.000	*0.001846*
6	24	300	74.0 ± 2.345	6	24	500	64.2 ± 3.271	6	24	700	60.4 ± 1.817	*0.002991*
*P*^*2*^ *= 0.0001818*				*p = 0.0002108*				*p = 0.0001506*				
Flow	RR	TV	FiO2	Flow	RR	TV	FiO2	Flow	RR	TV	FiO2	*p*
8	8	300	97.6 ± 0.548	8	8	500	94.4 ± 0.894	8	8	700	91.8 ± 1.483	*0.00244*
8	12	300	92.8 ± 2.950	2	12	500	86.8 ± 1.643	8	12	700	82.2 ± 1.924	*0.003*
8	16	300	88 ± 2.828	8	16	500	79.6 ± 0.548	8	16	700	76.8 ± 1.924	*0.003485*
8	20	300	84 ± 0.707	8	20	500	75 ± 2.345	8	20	700	73 ± 2.345	*0.00454*
8	24	300	81.4 ± 0.548	8	24	500	72 ± 2.000	8	24	700	70.2 ± 2.280	*0.00477*
*p = 0.0002186*				*p = 0.0001671*				*p = 0.0002253*				
Flow	RR	TV	FiO2	Flow	RR	TV	FiO2	Flow	RR	TV	FiO2	*p*
10	8	300	98.6 ± 0,548	10	8	500	97.4 ± 0.894	10	8	700	95.2 ± 1.095	*0.004186*
10	12	300	96 ± 1.225	10	12	500	90.8 ± 0.837	10	12	700	88.6 ± 1.517	*0.003444*
10	16	300	92.4 ± 3.050	10	16	500	85.2 ± 0.837	10	16	700	82.8 ± 1.483	*0.002752*
10	20	300	89.2 ± 1.304	10	20	500	82.2 ± 1.303	10	20	700	79 ± 1.871	*0.003252*
10	24	300	86.2 ± 0.837	10	24	500	78.2 ± 1.095	10	24	700	75.8 ± 1.924	*0.004176*
*p = 0.0002117*				*p = 0.0001203*				*p = 0.0001669*				

1. The units of flow, RR and TV are L/min, breaths/min, and ml

2. The significance of K_W test for comparison of different RR under controlled TV and flow

3. The significance of K_W test for comparison of different TV under controlled TV and flow

**Fig. 2:**
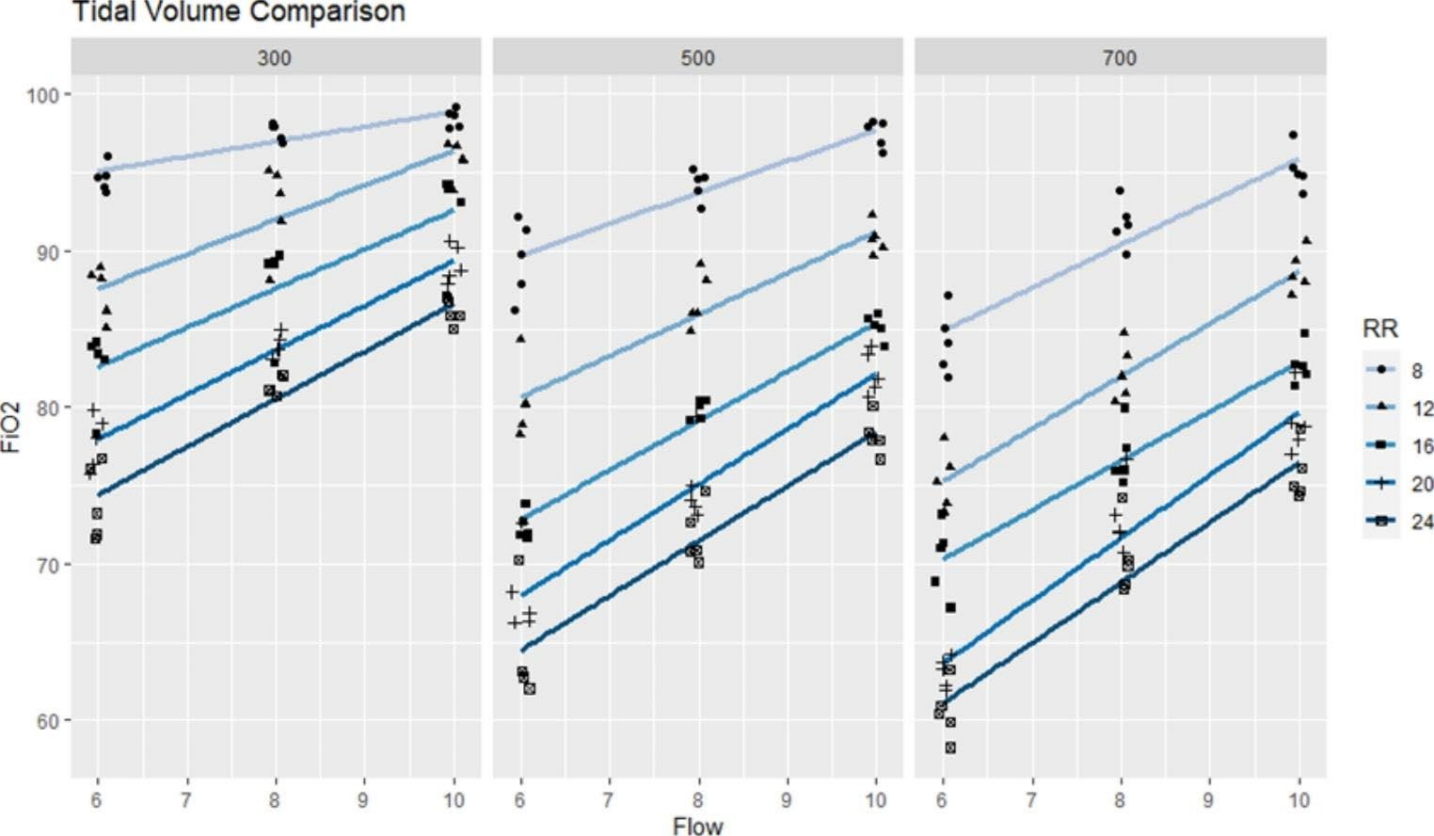
Respiratory rate and oxygen flow rate under fixed tidal volume. Regression lines are shown according to different RR. Data points are adjusted slightly, jittered to reveal all data

Comparisons between different RR and oxygen flow, when TV was controlled, are shown in Fig. 2. As RR increased, the measured FiO_2_ showed a statistically significant difference (p < 0.05) with low, normal, and high TV settings. However, the difference did become smaller under high oxygen flow rate. At the same RR, a lower FiO_2_ was recorded when a larger TV was set.

**Fig. 3:**
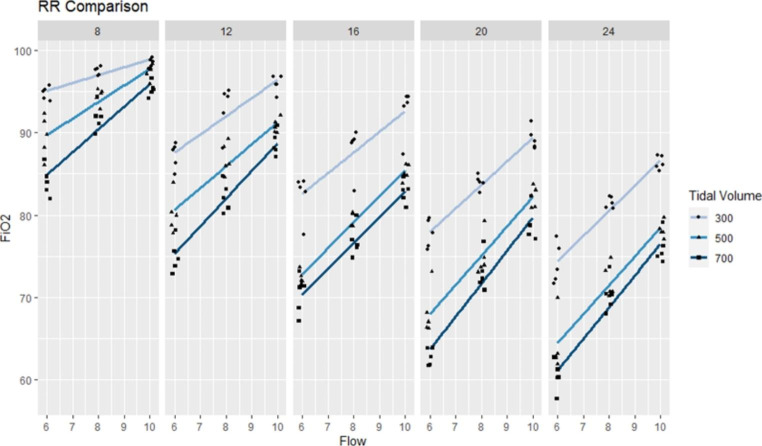
Tidal volume and oxygen flow rate under fixed respiratory rate. Regression lines are shown under different tidal volume. Data points are adjusted slightly, jittered to reveal all data

Figure 3 depicts comparisons of different TVs and oxygen flow when RR was controlled. Similar results were observed as the measured FiO_2_ showed significant differences with low to high RR settings, as TV increased. At the same TV, a lower FiO_2_ was recorded when a higher RR was set.

**Fig. 4:**
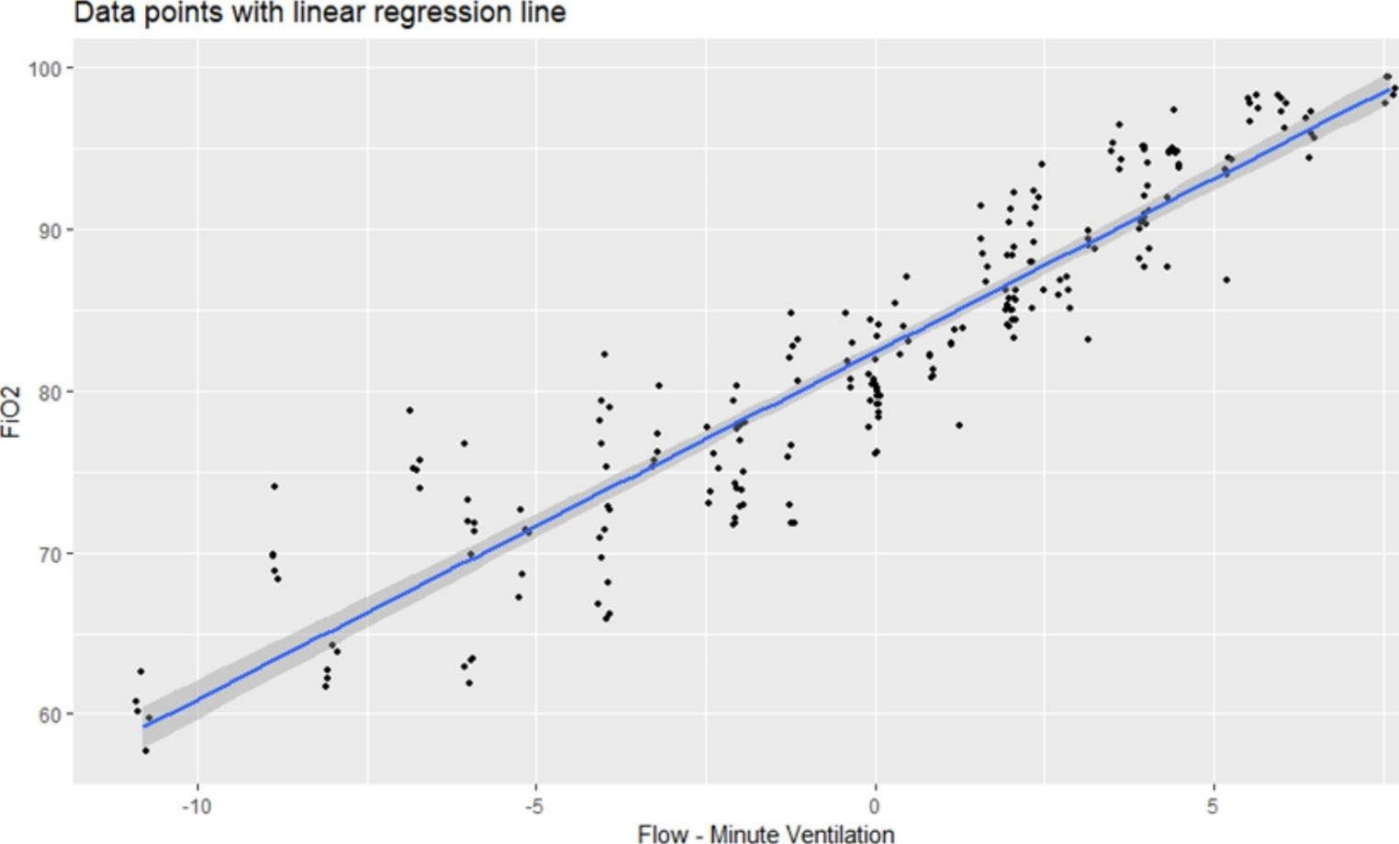
All data points are shown with the y axis as FiO2, and X axis being the difference in oxygen flow rate and minute ventilation. A linear regression line is plotted, showing good correlation. Data points are adjusted slightly, jittered to reveal all data

To further confirm our hypothesis, all samples are plotted in Fig. 4 with the X-axis representing the difference in oxygen flow and minute ventilation (product of TV and RR), and Y-axis representing FiO_2_. Thereafter, a linear regression line was drawn over the sample dots, demonstrating a highly correlated relationship. The relationship was tested using a linear regression model, with an R^2^ value of 0.86. The produced Eq. 1 is listed as follows:

Equation 1 $$Fi{O_2} = 82.373 + 2.146\left( {Oxygen\;flow\left( {L/min} \right)-minute\;ventilation} \right)$$

FiO_2_ = 82.373 + 2.146(oxygen flow(L/min)–minute ventilation) (Eq. 1.)

In order to find the best model for predicting FiO_2_, we ran a multiple linear regression model using TV, RR, and oxygen flow as coefficients, separately. The result was generated with an even better R^2^ value of 0.92. The produced Eq. 2 is listed as follows:

Equation 2 $$\eqalign{Fi{O_2} & = 92.033-0.026 \times TV\left( {ml} \right) \cr & -1.252 \times RR\left({breaths/minute} \right) \cr &+ 2.900 \times oxygen\,flow\left( {L/min} \right)}$$

FiO_2_ = 92.033–0.026 × TV(ml) – 1.252 × RR(breaths/minute) + 2.900 × oxygen flow(L/min) (Eq. 2.)

## Discussion

Our experiment demonstrated that, with a SentriO Oxy™ mask, variations in oxygen flow, TV, and RR influenced the delivery of oxygen concentrations in a lung simulation model. Although data such as SaO_2_ or PaO_2_ could not be obtained as in previous studies with human participants [13], we accurately measured FiO_2_ on the manikin model, which is difficult to observe in spontaneously breathing patients. Unlike previous studies that used the manikin simulation system to test face masks [14], we measured the FiO_2_ at the carina rather than in the oral cavity, which more accurately represents the oxygen content that can be utilized by patients. The small SD of each condition confirmed the stability and reproducibility of the measurements in our model, which is shown in Table 1. Based on our findings, the FiO_2_ measured by a smaller TV, lower RR, or higher oxygen flow revealed a higher value. In addition, we developed equations for clinicians’ reference. Our hypothesis that FiO_2_ is proportional to the difference between oxygen flows and minute ventilation was confirmed, and is clearly demonstrated by Eq. 1.

Patients undergoing sedative anesthetic procedures may encounter drug-induced respiratory depression, with apneic and sometimes hypoxemic episodes occurring frequently [15–17]. Breathing patterns may become shallower and slower, resulting in a decrease in minute ventilation. The minute ventilation of a normal adult during wakefulness is around 6–8 L/min, and decreases further during sleep [18]. Our settings involved minute ventilation ranging from 2.4 to 16.8 L/min, which included combinations from hypoventilation to hyperventilation. The difference between oxygen flow and minute ventilation was relatively larger during hypoventilation, resulting in a higher oxygen fraction. Moreover, during hyperventilation, oxygen was diluted with room air in the inspiratory phase, thus producing a lower oxygen fraction.

SentriO Oxy™ masks are equipped with an oxygen reservoir unit, which has the same effect on oxygen concentration as non-rebreathing masks. However, traditional non-rebreathing masks require a flow rate of 10 to 15 L/min to achieve FiO_2_ values between 0.6 and 0.8 [1, 2]. SentriO Oxy™ masks, on the other hand, claim to provide comparable results even on low-flow (5–10 L/min) oxygen. The results of our experiment supported the claim, with FiO_2_ ranging from approximately 0.6 to nearly 1.0 at various respiratory settings. These results were highest among those from other research, where different oxygenation equipment has been used on test lung models; the achieved FiO_2_ were less than 0.4, 0.6, and 0.8 when using low-flow nasal cannula, various masks, and specialized designed oral bite blocks under hypoventilation, respectively [11, 19, 20]. This indicates that the SentriO Oxy™ masks may be used in anesthetic procedures requiring oxygenation devices.

According to our calculations, the prediction formulas are suitable for adult patients. Our study demonstrated that TV, RR, and oxygen flows are all significant predictors affecting FiO_2_. Clinicians may use Eq. 2 to predict the most accurate FiO_2_, or Eq. 1 for a quick estimation. For example, according to Eq. 1, it is safe to estimate that FiO_2_ will almost surpass 0.8 under a hypoventilation scenario when oxygen flow rate is 6 L/min. This value is comparable with patients’ minute ventilation under anesthetic circumstances.

Our results also provide a reminder about the variability of the FiO_2_ delivered via SentriO Oxy™ masks. Clinicians must take the patients’ physiological conditions into account, such as alveolar PO_2_ or physiological shunt, to reduce the risk of hypoxemia.

### Limitations

The most significant limitation of our study is that a manikin head with a test lung model was used rather than actual human participants. However, the manikin model was easily standardized and reproducible, and the head model was also developed using adult 3D images for intubation training. Moreover, spontaneous breathing was simulated using mechanical test lungs, enabling the researchers to mimic real-life scenarios. However, when wearing SentriO Oxy™ masks, it is not possible for the clinician to directly measure the patient’s tidal volume or respiratory rate. Without this information, applying the specific equations may be difficult. Another limitation was that this study did not investigate the rebreathing phenomenon of carbon dioxide in this low flow oxygenation system. Hence, further studies are required for better clinical implications.

When a patient is anesthetized, airway obstruction may occur. However, our model did not include airway obstruction simulation, and the masks will not maintain a patent airway by themselves. Hence, even if they are not anesthesiologists, clinicians must be aware of the possibility of airway obstruction and be prepared to deal with such events [21].

## Conclusion

This bench study successfully demonstrated the stability and reproducibility of FiO_2_ measurements obtained through a manikin-test lung simulation system using SentriO Oxy™ masks. It was determined that parameters such as TV, RR, and oxygen flow were significant predicting factors affecting FiO_2_. In addition, a smaller TV, a lower RR, or a higher oxygen flow could result in a higher FiO_2_. Our experiment proposed two prediction equations considering oxygen flow rate, TV, and RR. The findings of our study may provide clinicians with information regarding the use of SentriO Oxy™ masks during sedative anesthetic procedures with a low oxygen flow rates.
